# Contamination of Phthalate Esters (PAEs) in Typical Wastewater-Irrigated Agricultural Soils in Hebei, North China

**DOI:** 10.1371/journal.pone.0137998

**Published:** 2015-09-11

**Authors:** Yuan Zhang, Qiong Liang, Rutai Gao, Haobo Hou, Wenbing Tan, Xiaosong He, Hui Zhang, Minda Yu, Lina Ma, Beidou Xi, Xiaowei Wang

**Affiliations:** 1 School of Resource and Environmental Science, Wuhan University, Wuhan 430079, China; 2 Beijing Key Laboratory of New Technique in Agricultural Application, College of Plant Science and Technology, Beijing University of Agriculture, Beijing 102206, China; 3 State Key Laboratory of Environmental Criteria and Risk Assessment, Chinese Research Academy of Environmental Sciences, Beijing 100012, China; Institute for Sustainable Plant Protection, C.N.R., ITALY

## Abstract

The Wangyang River (WYR) basin is a typical wastewater irrigation area in Hebei Province, North China. This study investigated the concentration and distribution of six priority phthalate esters (PAEs) in the agricultural soils in this area. Thirty-nine soil samples (0–20 cm) were collected along the WYR to assess the PAE residues in soils. Results showed that PAEs are ubiquitous environmental contaminants in the topsoil obtained from the irrigation area. The concentrations of Σ_6_PAEs range from 0.191 μg g^−1^ dw to 0.457 μg g^−1^ dw with an average value of 0.294 μg g^−1^ dw. Di(2-ethylhexyl) phthalate (DEHP) and di-*n*-butyl phthalate (D*n*BP) are the dominant PAE species in the agricultural soils. Among the DEHP concentrations, the highest DEHP concentration was found at the sites close to the villages; this result suggested that dense anthropogenic activities and random garbage disposal in the rural area are possible sources of PAEs. The PAE concentrations were weakly and positively correlated with soil organic carbon and soil enzyme activities; thus, these factors can affect the distribution of PAEs. This study further showed that only dimethyl phthalate (DMP) concentrations exceeded the recommended allowable concentrations; no remediation measures are necessary to control the PAEs in the WYR area. However, the PAEs in the topsoil may pose a potential risk to the ecosystem and human health in this area. Therefore, the exacerbating PAE pollution should be addressed.

## Introduction

Phthalate esters (PAEs) are widely used in different products, including industrial and commercial products, such as pharmaceutical pills, detergents, packaging, paints, and pesticides [1–2]. PAEs have caused considerable concern over their widespread distribution and potentially hazardous impact on the environment. The global production is more than 8.0 million tons of PAEs annually [3]. High PAE levels have been explained in terms of various environmental matrices [4–9]. The Environmental Protection Agency (EPA) of the USA and the Chinese State Environmental Protection Administration have also considered PAEs as environmental priority pollutants. Three phthalates (dimethyl phthalate (DMP), di-*n*-butyl phthalate (D*n*BP), di-*n*-octyl phthalate (D*n*OP)) are also in the black list of China’s water priority pollutants, as identified by the Chinese National Environmental Monitoring Center.

The Wangyang River (WYR) is situated in Shijiazhuang City, the capital of Hebei Province in North China. The 85 km long river is the tributary of the Ziya River, which is affiliated to the Haihe River Basin. Beijing and Tianjin also belong to the region of the Haihe River Basin. The industrial effluents from petrochemical, electronic, and pharmaceutical factories are discharged into the WYR after these effluents are subjected to secondary (biological) treatment. The river water has been used to irrigate farmland soils for more than 20 years [10]. The predominant cropping system is winter wheat (*Triticum aestivum* L.)-summer maize (*Zea mays* L.) rotation that covers 60% of the arable land in this area. However, the contribution of wastewater to the occurrence of PAEs in agricultural soils through irrigation and the biological effects of this contaminant have not yet been determined in the rivers receiving treated and untreated wastewater as the main water sources. Furthermore, the accumulation of PAEs in agricultural soils may contaminate vegetables and food chains; this phenomenon results in direct or indirect human exposure [8]. Moreover, the leaching, evaporation, and migration of PAEs are possible sources of atmospheric or groundwater contamination [3].

The environmental problem in this region has been extensively investigated by scientific and regulatory communities. In this study, the distribution and characterization of PAEs in WYR were determined; the potential PAE sources were also evaluated. Moreover, the effects of the PAE contamination on the soil microbial activities in the wastewater-irrigated fields were analyzed. This study provides the missing data on the distribution of PAEs in the Haihe River Basin and contributes to the assessment of the ecological risk of these pollutants.

## Materials and Methods

### Sampling

**Fig 1:**
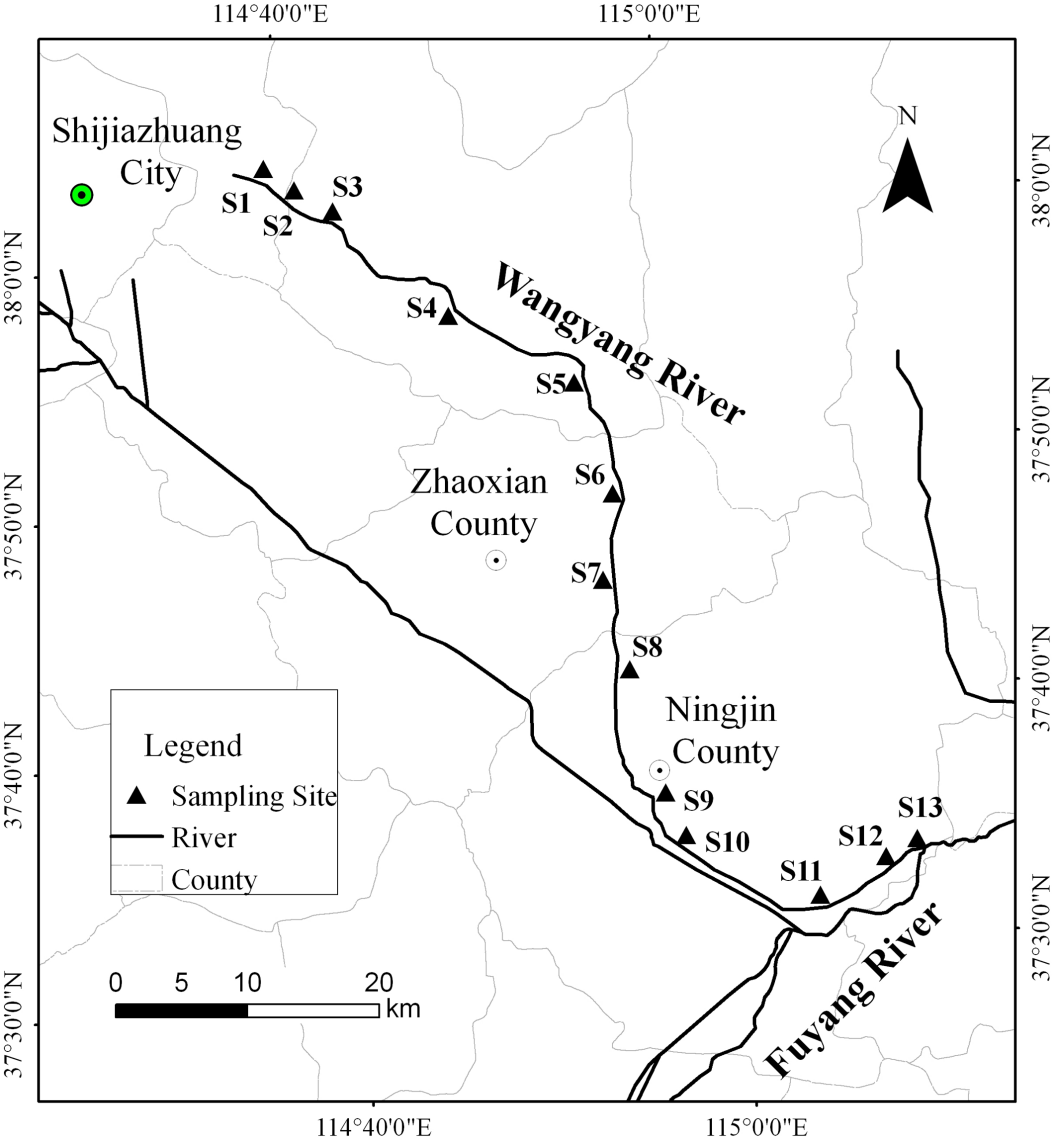
Sampling sites in Wangyang River area.

No specific permissions were required for sampling locations/activities. The field studies did not involve endangered or protected species. The solid and liquid samples were collected in June 2013. The soil samples irrigated with river water were collected from thirteen sites along the WYR (Fig 1). The topsoil (0–20 cm) samples were collected in triplicate from each site with a 10 cm diameter soil core sampler. The field-moisture samples were passed through a 2 mm sieve to remove stone and plant residues and then divided into two parts. One part was frozen for biochemical analyses. The other part was air-dried at room temperature and ground for physical and chemical analyses. The river water and sediment samples were also collected from the thirteen sites in the WYR. Three water samples and four sediment samples were obtained at each site. The water samples were collected with a stainless steel bucket and stored in glass bottles at 4°C. Surface sediment samples (0–5 cm) were collected with a grab sampler. All of the samples were transported to our laboratory in an ice box and treated in 7 days.

### Chemicals and materials

Reference PAEs including dimethyl phthalate (DMP), diethyl phthalate (DEP), di-*n*-butyl phthalate (D*n*BP), butyl benzyl phthalate (BBP), di-*n*-octyl phthalate (D*n*OP), di (2-ethylhexyl) phthalate (DEHP) were purchased from AccuStandard Inc., USA. All solvents used for sample processing and analyses were chromatographic grade from Dikma Technologies Inc., USA.

### Sample pretreatment

The water samples (1 L) were filtered at a flow rate of 5 mL min^−1^ by using solid-phase extraction (SPE) cartridges (HLB cartridges, 500 mg per 6 mL; Waters) pre-conditioned with 5 mL of dichloromethane, 5 mL of methanol, and 5 mL of ultra-pure water. The PAEs were eluted from the cartridge by using 10 mL of dichloromethane and collected in 10 mL glass tubes with stoppers. The eluate was evaporated under a stream of purified N_2_ until the final volume of 1 mL was obtained for GC-MS analysis and quantified using the external standard method.

The sediment and soil samples were dried using a freeze-drying apparatus and ground with a 100-mesh sieve. In brief, 5 g of the dry sample was combined in a 250 mL conical flask with an acetone-dichloromethane mixture (1/2, v/v) as the extraction solvent for 20 min of ultrasonic extraction. After the extracts were allowed to stand for several minutes, the supernatants were transferred to 50 mL centrifuge tubes. The final extraction volume was approximately 20 mL in three extraction cycles. The extracts were completely condensed to approximately 10 mL and then subjected to SPE for cleanup and concentrated to 1 mL for further analysis.

### Instrumental analysis

The concentrations of PAEs in water, sediment, and soil samples were analyzed with an Agilent 6890N gas chromatograph with a 5973C mass selective detector (GC/MSD), equipped with an Agilent 7683B automatic liquid sampler and a DB-5 MS capillary column (30 m, 0.25 mm i.d., 0.25 μm film thickness). Helium was used as the carrier gas with a column flow rate of 1.2 mL min^−1^ in a constant-flow mode. The injector, ion source, and transfer line temperatures were set at 280, 290, and 260°C, respectively. The GC oven temperature was programmed from 70°C (2 min) to 130°C (1 min) at 20°C min^−1^, increased to 240°C at 5°C min^−1^, and held constant for 1 min; the temperature was further increased to 260°C at 5°C min^−1^ and held constant for 1 min; the temperature was subsequently increased to 270°C at 5°C min^−1^ and held constant for 1 min; afterward, the temperature was increased to 280°C at 10°C min^−1^ and held constant for 7 min. The electron impact energy was set at 70 eV, and 2 μL of each sample was injected in the splitless mode. The PAEs were analyzed in the selected ion monitoring mode.

### Quality control and assurance

The blanks covering the entire analytical procedure, including extraction and GC analysis, were analyzed. The PAE levels in water, sediment, and soil blanks were not detected or were much lower than the detection limits of the respective method; in particular, the PAE levels in water, sediment, and soil were 0.08–1.1 ng L^−1^, 0.12–1.9 ng g^−1^, and 0.13–2.1 ng g^−1^, respectively. The recovery rates of DMP, DEP, D*n*BP, BBP, D*n*OP, and DEHP were 103.47%, 91.93%, 109.58%, 98.65%, 84.81%, and 85.34%, respectively.

### Soil physico-chemical analysis

Soil pH was determined using a PHS-3C pH meter (REX Instrument Factory, Shanghai, China) in a 1:2.5 suspension in a 0.01 M CaCl_2_ solution. Soil electrical conductivity (EC) was measured using a DDS-307 conductivity meter (REX Instrument Factory, Shanghai, China) by mixing 10 g of soil with 50 mL of distilled water. Soil texture was evaluated via the pipette method [11]. The soil samples for soil organic C (SOC) measurements were pretreated with 0.5 M HCl to remove the carbonates and ball-milled [12]. The SOC concentration was determined through dry combustion (Analytik Jena, Germany).

Soil microbial biomass C (MBC) was determined via the chloroform fumigation–extraction method on fresh soil samples [13]. The fumigated and unfumigated soils were shaken for 30 min with 0.5 M K_2_SO_4_ and analyzed for C by using a Multi 3100N/C TOC analyzer (Analytik Jena, Germany). To obtain the soil MBC, we divided the difference of the extractable C between fumigated and unfumigated soils by a conversion factor of 0.45 [14]. The unfumigated samples were used to estimate the background dissolved organic C (DOC) values [15]. Permanganate oxidizable C (KMnO_4_-C) was estimated in accordance with a previously described method [16]. Catalase activity was determined via a titration method [17]; the activities of dehydrogenase, alkaline phosphatase, β-glucosidase, arylsulphatase, and urease were determined in accordance with previously described methods [18]. The dehydrogenase activity was evaluated with triphenyltetrazolium chloride as a substrate; the samples were incubated for 24 h at 37°C. The activities of β-glucosidase, alkaline phosphatase, and arylsulphatase were determined with *p*-nitrophenyl-β-d-glucopyranoside, *p*-nitrophenyl phosphate, and *p*-nitrophenyl sulfate as substrates, respectively; the samples were incubated for 1 h at 37°C for the analyses. The urease activity was evaluated with urea as a substrate; the samples were incubated for 2 h at 37°C and examined through steam distillation. All of the measured parameters were calculated the basis of dry matter.

### Statistical analysis

Analytical results were calculated on the basis of the oven-dried (105°C) weight of soil. Data were analyzed using SPSS 19.0 for Windows. The PAE concentrations among the sampling sites were analyzed through one-way ANOVA to evaluate the effects of wastewater irrigation. Differences were evaluated and considered significant at *p* < 0.05.

## Results and Discussion

### Levels of PAEs in water, sediments, and agricultural soils of the WYR

The individual concentrations of the six priority PAEs in water, sediments, and agricultural soils of the WYR are shown in Table 1. PAEs were detected in all of the river water samples, thereby indicating that PAEs are ubiquitous environmental contaminants in the WYR. The total concentrations of the six PAEs in the river water ranged from 2.42 μg L^−1^ to 6.67 μg L^−1^ with an average value of 4.01 μg L^−1^. These results were comparable to those of several previous studies; the levels of the compounds are often of the same order of magnitude as those found in other countries (Table 2) [4,19–22]. Nevertheless, a comparison with internal studies (Table 2) [23–25] indicated that these levels were below the average levels. This result may be related to the different sources of the river. Moreover, PAE contamination is currently low in irrigation water, but further studies should be conducted to trace the flow of PAEs in the WYR.

**Table 1:** Summary of the individual concentrations of PAEs in the WYR water, sediments, and agricultural soils (*n* = 39).

PAEs	River water^a^ (μg L^−1^)				Sediments^a^ (μg g^−1^ dw)				Soils^a^ (μg g^−1^ dw)			
	Range	Mean ± SD	Median	DF	Range	Mean ± SD	Median	DF	Range	Mean ± SD	Median	DF
DMP	0.31–1.87	0.64 ± 0.45	0.43	100	ND–0.067	0.020 ± 0.021	0.026	53.8	ND–0.034	0.024 ± 0.008	0.024	89.7
DEP	0.23–1.00	0.34 ± 0.15	0.28	100	ND–0.069	0.022 ± 0.021	0.032	56.4	0.023–0.034	0.026 ± 0.003	0.026	100
D*n*BP	0.32–3.65	0.95 ± 0.69	0.81	100	0.042–0.159	0.084 ± 0.033	0.072	100	0.035–0.054	0.045 ± 0.005	0.045	100
BBP	0.39–3.36	1.18 ± 0.68	1.06	100	ND–0.111	0.028 ± 0.029	0.034	61.5	ND–0.116	0.022 ± 0.025	0.029	51.3
DEHP	0.26–0.94	0.48 ± 0.15	0.43	100	0.161–0.465	0.307 ± 0.078	0.304	100	0.066–0.263	0.143 ± 0.052	0.132	100
D*n*OP	0.31–0.65	0.40 ± 0.07	0.39	100	0.043–0.142	0.077 ± 0.021	0.077	100	ND–0.069	0.036 ± 0.019	0.041	82.1
Σ_6_PAEs	2.42–6.67	4.01 ± 1.16	3.74		0.367–0.729	0.536 ± 0.096	0.529		0.191–0.457	0.294 ± 0.060	0.287	

DF, detectable frequency (%). ND, concentration was lower than the MDL. dw, dry weight.

^a^ reported concentrations were corrected by subtracting the mean blank values.

**Table 2:** Concentrations of the six target PAEs in water compared with other studies (μg L^−1^).

Location	DMP	DEP	DBP	DEHP	DNOP	Reference, Year
Klang River Basin, Malaysia	ND–0.1	ND–0.2	0.8–4.8	3.1–64.3	ND–1.5	Tan [19], 1995
Velino River, Italy	—	ND–3.2	ND–44.3	ND–31.2	ND–11.3	Vitali et al. [4], 1997
Ebro River, Spain	ND	0.26	—	0.7	—	Penalver et al. [20], 2000
East London Port, South Africa	0.03–31.7	0.03–33.1	2.8–12.19	0.06–19.74	—	Fatoki and Noma [21], 2002
Dutch aquatic, Netherlands	0.05–0.19	0.07–0.23	0.07–3.1	0.9–5	< 0.01–0.08	Vethaak et al. [22], 2005
Guiyang, China	ND	ND–4.5	3.1–13.9	14.8–235	—	Seth et al. [23], 1999
Yellow River, China	ND–0.58	0.012–1.093	ND–26	0.35–24	ND–7.1	Sha et al. [24], 2007
Haihe River, China	—	—	0.35–40.68	3.54–101.1	—	Chi [25], 2009
This article	0.31–1.87	0.25–1.00	0.32–3.65	—	0.26–0.84	

ND, concentration was lower than the MDL.

In the sediments, the total content of the six PAEs varied considerably, ranging from 0.367 μg g^−1^ to 0.729 μg g^−1^ based on dry weight (dw), with an average value of 0.536 μg g^−1^ dw (Table 1). Among the individual PAEs, D*n*BP, DEHP, and D*n*OP with the respective mean concentrations of 0.084, 0.307, and 0.077 μg g^−1^ dw were present in all of samples; by contrast, the other PAEs exhibited relatively low concentrations and detectable frequencies. The concentrations of the six PAEs in sediments samples from WYR were lower than those from the Songhua River in China (Σ_6_PAEs ranged from 0.381 μg g^−1^ to 2.044 μg g^−1^) [2] and the suburban southern Jiangsu River (Σ_6_PAEs ranged from 2.3 μg g^−1^ to 80.1 μg g^−1^) [26]. The differences in the PAE profiles of sediments from different regions may reflect the predominantly utilized PAE formulation. Therefore, the sources and modes of transfer to the river require investigation in future studies [26].

The sum of the concentrations of the six PAEs in the agricultural soils along the WYR considerably varied, ranging from 0.191 μg g^−1^ dw to 0.457 μg g^−1^ dw, which are within the less stringent grade II limits of PAEs (10 μg g^−1^ dw) in arable soils recommended by the Environmental Quality Standard for soil in China (GB-15618-2008) [27]. Compared with the PAE concentrations in the rural soils of the Yellow River Delta (Σ_6_PAEs ranged from 0.716 μg g^−1^ dw to 3.25 μg g^−1^ dw) [7] and those in black soils of northeast China (Σ_16_PAEs ranged from 1.37 dw to 4.90 dw) [9], the results of the present study were relatively low. This difference may be attributed to the different cropping systems and field management practices. The predominant cropping system in the WYR area is winter wheat-summer maize rotation, with a large vegetable or orchard land area in the Yellow River Delta and black soil in northeastern China; these conditions may have introduced more PAE contaminants to the topsoil by intensive management practices, such as greenhouses for vegetables, plastic mulching for cotton, apple bagging, and pesticide application [7]. In the present studied area, the wastewater discharged into the river mainly originated from the effluent of wastewater treatment plants (WWPT) in Shijiazhuang, which is located in the upstream region of the WYR; the said wastewater was used for irrigation of this agricultural field [28]. Irrigation with wastewater from domestic and industrial sources has been known to contribute significantly to the amount of toxic contaminants, such as heavy metals, PAEs, and polychlorinated biphenyls, in the soils [29–30], even after the samples were treated by the WWPT. Thus, the wastewater irrigation might be an important source of PAE content in agricultural soils. This observation is supported by the study of Niu et al. [31], who found that application of plastic films, wastewater irrigation, and fertilizers were the predominant sources of PAEs in arable soils across China.

### PAE congener profiles and distribution in agricultural soils

**Fig 2:**
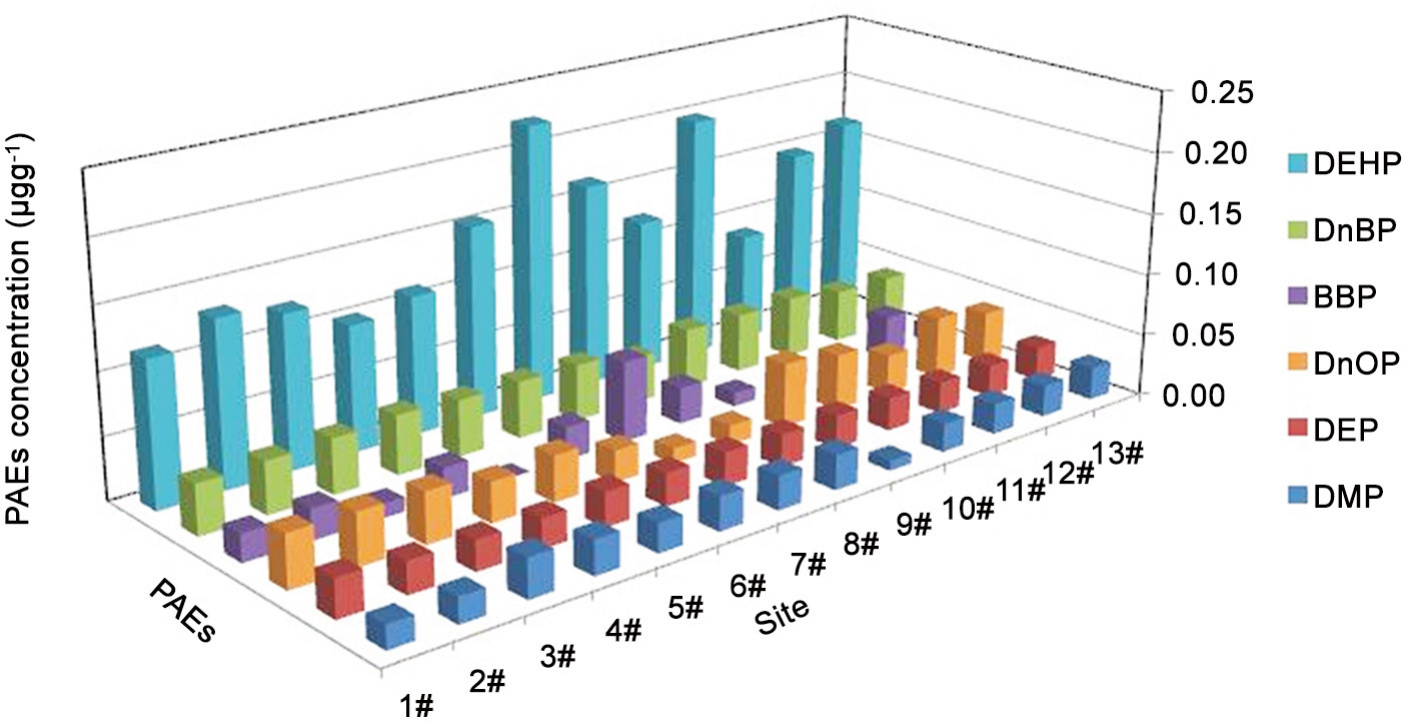
Concentrations of 6 PAEs congeners in the agricultural soils along the WYR.

The PAE congener profiles and distribution in agricultural soils from different areas along the WYR are shown in Table 1 and Fig 2. DEP, D*n*BP, and DEHP were present in all of the agricultural soil samples along the WYR; the average concentrations of the three substances were 0.0260, 0.0450, and 0.143 μg g^−1^ dw, respectively (Table 1); conversely, the detection frequencies of other PAEs showed a decreasing pattern: DMP (89.7%), D*n*OP (82.1%), and BBP (51.3%). The dominant PAE species in the agricultural soils was DEHP that accounted for 48.6% of the total PAEs, followed by D*n*BP, which accounted for 15.3%. This trend is inconsistent with that described in previous reports, which indicated that DEHP and D*n*BP are the dominant components of the PAE distribution pattern in the environment [22,32–34]. In addition, DEHP and D*n*BP are among the most commonly produced PAEs used as plastic additives [4]. Furthermore, the dominant PAE species in the river water was BBP (Table 1), which was inconsistent with those in sediment and agricultural soils. This discrepancy may be possible because the species and concentrations of the PAEs detected in the river water were more dependent on the instantaneous concentrations in the wastewater than on other parameters; by contrast, the concentrations of the PAEs in the sediment and agricultural soils mainly resulted from long-term accumulation.

The high proportion of DEHP and D*n*BP in the soils was similar to the results detected in the sediment samples (Table 1), thereby indicating the same accumulation characteristics of the PAEs in the two environmental matrices. This result suggested that the sediments may be a potential pollution source of PAEs in the agricultural soils. A part of the sediments may be introduced using pumps during irrigation and accumulate in the topsoil of farmlands because of the shallow depth of the river. This mechanism of irrigation is similar to that in sediment-laden water, which may introduce organic matter and contaminants to soils [35]. The increased DEHP and D*n*BP levels in agricultural soils derived from wastewater irrigation in China have been discussed. Zeng et al. [6] detected the PAE levels in the agricultural soils from the peri-urban areas of Guangzhou City; these soils are heavily polluted by long-term industrial wastewater irrigation and sludge application; DEHP and D*n*BP concentrations ranged from 0.107 μg g^−1^ dw to 29.4 μg g^−1^ dw and from 0.009 μg g^−1^ dw to 2.74 μg g^−1^ dw, respectively. Kong et al. [36] studied the diversity of PAEs in suburban agricultural soils under wastewater irrigation for more than 40 years in Tianjin; their group showed DEHP and D*n*BP concentrations of 0.026–4.17 and 0.007–0.285 μg g^−1^ dw, respectively. Wang et al. [37] studied the accumulation and distribution of phthalate ester residues in soils from the Sanjiang Plain (3.1–110.7 ng g^−1^ dw for DEHP, 1.5–133.6 ng g^−1^ dw for D*n*BP); the residual concentrations of PAEs in the paddy field were possibly caused by the surface coverage of irrigation water. Compared with the concentrations inthe other areas, the concentrations of DEHP and D*n*BP in the agricultural soils along the WYR (0.066–0.263 μg g^−1^ dw for DEHP, 0.035–0.054 μg g^−1^ dw for D*n*BP) were relatively low. These trends may be attributed to the dilution effect of the PAEs in the river water, and this effect could decrease the PAE concentrations in the irrigation water and reduce the accumulation rate in soils.

The spatial distribution of PAEs showed that the DEHP concentration fluctuated in the agricultural soils along the WYR, whereas other PAEs showed no significant differences among the different sites (Fig 2). The highest DEHP concentration was found at site 7 (0.225 μg g^−1^ dw), followed by the DEHP concentration at site 10 (0.199 μg g^−1^ dw); both sites were immediately close to villages, where a significant amount of agricultural waste and garbage (including plastic films, wrapping materials, pesticide bottles, and construction waste) were discarded at the bank of the river. Garbage and its leachate have been reported as important sources of PAEs in the surface water and soils [38]. Consequently, the high concentrations of PAEs in the topsoil probably resulted from the dense anthropogenic activity and random dumping of garbage, especially in the rural areas. This result is consistent with that of Kong et al. [36], who found that the leachate from stacked wastes, including building materials and domestic garbage, may contribute a considerable amount of PAEs to the soil environment. Their group pointed out that disordered piling of waste on suburban soil should be cleaned and prohibited in the future. Thus, the concentration and contribution of PAEs to the agricultural soils along the WYR were attributed to the application of wastewater, sewage sludge, plastic films, pesticides, and agricultural management practices; the concentration and contribution of PAEs were also caused by human activities and garbage pollution in rural areas. The PAEs in agricultural soils may remain on the soil surface or may be transferred to deeper soil layers; both conditions can cause long-term harmful effects on ecosystems and human health.

### Characteristics of agricultural soils

The distribution of PAEs is closely related to the nature of the soil; thus, we analyzed the soil components. The properties of the agricultural soils along the WYR, including the pH, EC, moisture content, clay content, soil organic carbon fractions, and enzyme activity are presented in Table 3. The soils along the WYR are weakly basic, with pH (CaCl_2_) values ranging from 7.57 to 7.90. The soil EC varied between 93 and 430, thereby reflecting the slightly salinity of the soils. The texture of the agricultural soils was silt loam or silt clay loam, and the clay content ranged from 14.3% to 39.8%. The average SOC content was 13.75 g kg^−1^, whereas the mean content of labile organic C in terms of DOC, MBC, and KMnO_4_-C was 0.120, 0.320, and 3.07 g kg^−1^, which accounted for 0.87%, 2.33%, and 22.33% of the SOC, respectively. The activity of alkaline phosphatase, arylsulphatase, β-glucosidase, urease, dehydrogenase, and catalase varied and depended on the different kinds of enzymes. The spatial distribution was similar to the gradient of labile organic C fractions, which were higher in the downstream soils than in the upstream soils (data not shown). Therefore, the availability of labile C is an important determinant of the enzyme activities in soils.

**Table 3:** Major characteristics of agricultural soils along the WYR (*n* = 39).

	Range	Mean ± SD	Median
pH_CaCl2_	7.57–7.90	7.71 ± 0.08	7.69
EC (μS cm^−1^)	93–430	201 ± 91	169
θ_m_ (g g^−1^)	0.14–0.28	0.21 ± 0.04	0.21
Clay (%)	14.3–39.8	23.6 ± 6.6	22.4
SOC (g kg^−1^ dw)	6.07–33.11	13.75 ± 8.14	10.12
DOC (g kg^−1^ dw)	0.07–0.17	0.12 ± 0.03	0.11
MBC (g kg^−1^ dw)	0.09–0.71	0.32 ± 0.16	0.27
KMnO_4_-C (g kg^−1^ dw)	1.13–5.25	3.07 ± 0.92	3.18
Alkaline phosphatase (μg PNP g^−1^ dw h^−1^)	234.8–723.2	521.4 ± 102.0	513.1
Arylsulphatase (μg PNP g^−1^ dw h^−1^)	13.3–132.1	64.8 ± 27.0	61.6
β-Glucosidase (μg PNP g^−1^ dw h^−1^)	20.9–234.5	127.5 ± 53.5	120.7
Urease (μg NH_4_^+^-N g^−1^ dw 2 h^−1^)	69.1–486.7	238.3 ± 105.0	215.7
Dehydrogenase (μg TPF g^−1^ dw 24 h^−1^)	96.8–317.4	213.0 ± 48.1	213.0
Catalase (mL (20 mM KMnO_4_) g^−1^ dw h^−1^)	0.42–1.29	0.72 ± 0.22	0.67

EC, electrical conductivity; θ_m_, soil water content; SOC, soil organic carbon; DOC, dissolved organic carbon; MBC, microbial biomass carbon; KMnO_4_-C, permanganate oxidizable carbon; dw, dry weight.

### Correlations of PAE concentrations with SOC fractions and soil enzyme activities

**Fig 3:**
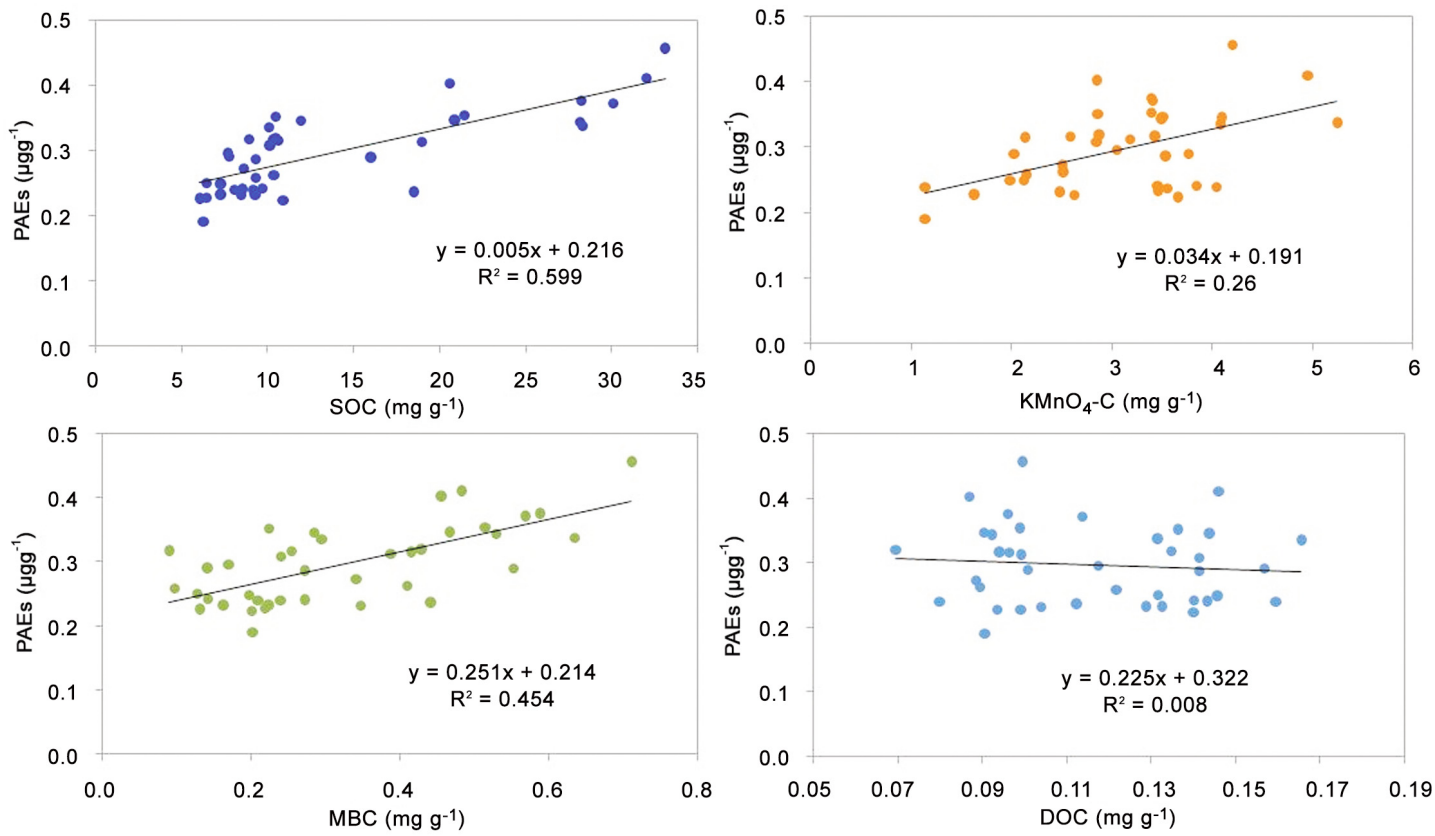
Correlations of PAEs concentrations with the soil organic carbon fractions in the agricultural soils along the WYR. SOC, soil organic carbon; KMnO_4_-C, permanganate oxidizable carbon; MBC, microbial biomass carbon; DOC, dissolved organic carbon.

The concentrations and distribution of PAEs in the soil could be influenced by various factors, such as the pH, clay content, organic matter, humic substances, and the microbial activity. PAEs also have significant effects on soil biological and microbiological properties because of their toxicity. The importance of soil organic matter has been shown in the mobility of hydrophobic organic contaminants in soils, such that PAEs are likely adsorbed by soil organic matter [7, 35]. Therefore, the SOC content could affect the PAE concentrations in the soil. The relationship was analyzed between the concentrations of the Σ_6_PAEs and the soil organic carbon fractions in the studied soil samples. Fig 3 shows that the correlation coefficient (*R* ^2^) was 0.559 (*n* = 39), thereby indicating a positive correlation between Σ_6_PAEs and SOC in the investigated soils. Similar results were reported by Zeng et al. [6] and Yang et al. [7]. The present results also show that the concentrations of Σ_6_PAEs were weakly and positively correlated with the MBC and KMnO_4_-C contents, with *R* ^2^ of 0.454 and 0.26, respectively. These trends suggested that labile SOC fractions may affect the distribution of PAEs in the studied area. However, the concentrations of Σ_6_PAEs were poorly correlated with DOC, with an *R* ^2^ of only 0.008 (*n* = 39; Fig 3). The low correlation may be attributed to the small size, highly labile nature, and mobility of DOC. However, other researchers previously showed that DOC may play an important role in the coexistence of PAEs and heavy metals in the sediments and enhance the PAE sorption to sediments [39].

**Fig 4:**
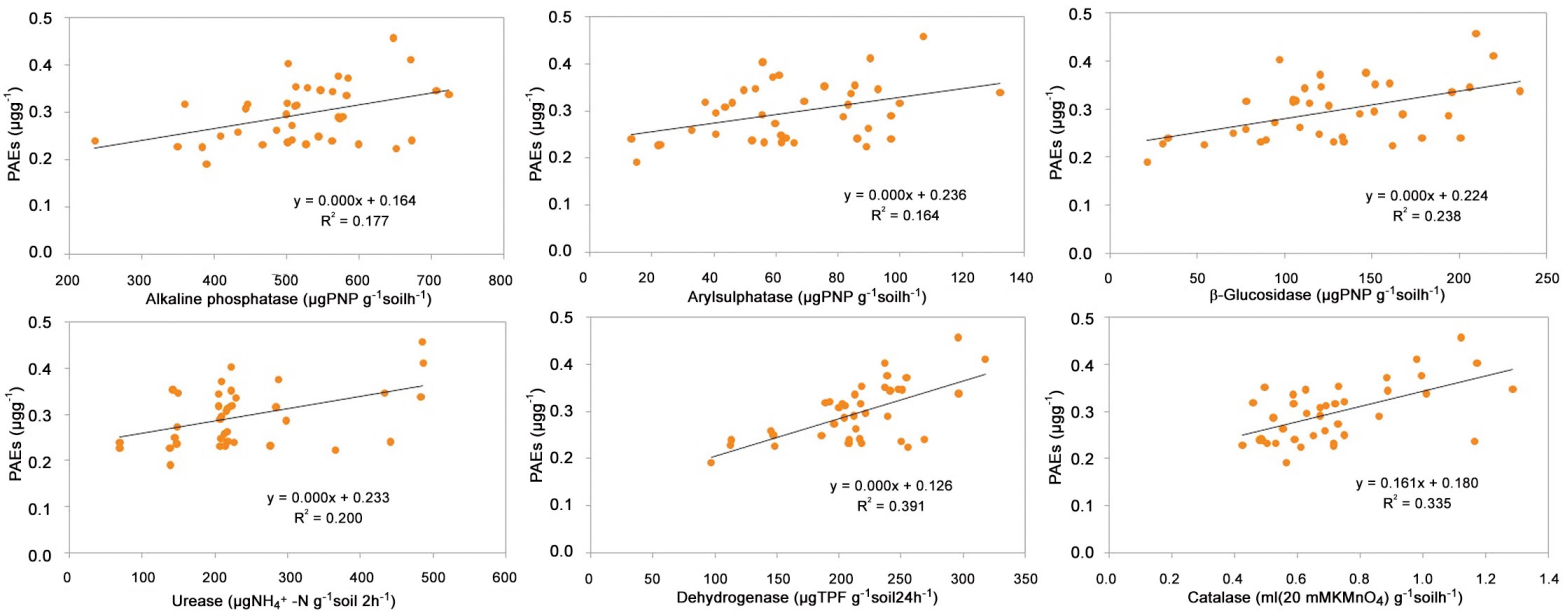
Correlations of PAEs concentrations with the soil enzyme activities in the agricultural soils along the WYR.

Soil enzyme activities can respond to changes more quickly in soil management, fertilization, and environmental pollution than other soil properties [40]. Thus, the soil enzyme activities can be used as soil quality and contamination indicators. The relationship between Σ_6_PAE concentrations and soil enzyme activities is shown in Fig 4. The weakly positive correlations of the Σ_6_PAEs and soil enzyme activities (*R* ^2^ ranged from 0.164 for arylsulphatase to 0.391 for dehydrogenase, *n* = 39) indicated that the existence of the PAEs in the soils did not decrease microbial activities in the studied area. Although PAEs are considered toxic to microorganisms [1, 41], their inhibitory effects on microbial activities were weaker than the promoting effects of soil organic matter in our study.

### Potential risk assessment of the agricultural soils

The six PAEs in the present study have attracted much attention worldwide and are listed as priority pollutants and endocrine-disrupting compounds by the US EPA, Chinese National Environmental Monitoring Center, and other regulatory bodies. The concentrations of PAEs recorded in the present study were used to assess the potential risk from the agricultural soils along the WYR. The PAE concentrations were compared with the soil cleanup guidelines used in New York, USA because of the shortage of local standards for soil pollution identification [42].

In the agricultural soil samples from the WYR area, only the DMP levels exceeded the recommended allowable concentrations (0.02 μg g^−1^ dw), which is 75% more than the standard value. However, the DMP concentrations in the present study were far below the recommended soil cleanup levels set by the USA standards (2.0 μg g^−1^ dw). The concentrations of the other PAEs in the soil samples were below the recommended allowable concentrations (0.071 μg g^−1^ dw for DEP, 0.081 μg g^−1^ dw for D*n*BP, 1.215 μg g^−1^ dw for BBP, 4.35 μg g^−1^ dw for DEHP, and 1.20 μg g^−1^ dw for D*n*OP). Therefore, no remediation measures are needed in terms of the PAEs in the WYR area. However, the relatively lower concentrations of PAEs may pose a potential long-term risk to the ecosystem and human health through the food chain [35]. Therefore, further studies should be conducted on the basis of the possible biological magnifications of PAEs.

## Conclusion

This study reports the concentrations and distribution of PAEs in the agricultural soils from the WYR basin. The six priority PAEs were ubiquitous in the topsoil of the wastewater irrigation areas; the total concentrations range from 0.191 μg g^−1^ dw to 0.457 μg g^−1^ dw. As the dominant PAEs in agricultural soils, DEHP and D*n*BP accounted for 63.9% of the total PAE concentration. Positive correlations were observed between the PAE concentrations and the soil organic carbon fractions and soil enzyme activities; therefore, these factors can affect the distribution of PAEs. The predominant cropping system of the study area was wheat-maize rotation; plastic films are applied to a low extent during agricultural management. The wastewater irrigation and stacked domestic garbage were considered as the major sources of PAE contaminants in this area. Compared with the results of other studies, our findings show that the agricultural soils in the WYR wastewater irrigation area were weakly polluted by PAEs; despite this finding, no remediation measures were required. However, the PAE pollution in this area should be addressed because this phenomenon may pose a potential long-term risk to the ecosystem and human health.
